# Effects of Microplastics and Cd/Pb Co-Contamination on Tobacco (*Nicotiana tabacum* L.) Growth and Antioxidant Systems

**DOI:** 10.3390/plants15111755

**Published:** 2026-06-05

**Authors:** Shengxue Guan, Yiwen Hu, Ke Jiang, Taoze Liu, Jiegang Liu, Hui Wang, Zhanghong Wang

**Affiliations:** 1 College of Eco-Environmental Engineering, Guizhou Minzu University, Guiyang 550025, China; 2 Engineering Research Center of Green and Low-Carbon Technology for Plastic Application, Guizhou Minzu University, Guiyang 550025, China

**Keywords:** antioxidant system, co-contamination, growth, heavy metal, microplastic, tobacco

## Abstract

The coexistence of microplastics (MPs) and heavy metals (Cd, Pb) in agricultural soils has become a global environmental and ecological risk. In this study, a pot experiment was conducted to investigate the effects of different concentrations of polyethylene (PE) microplastics and combined Cd/Pb contamination on the growth and development, heavy metal accumulation, and antioxidant system of tobacco (*Nicotiana tabacum* L. cv. Yunyan 87). The results showed that low-dose PE and low concentrations of heavy metals had minor impacts on tobacco growth and the antioxidant system; in contrast, high-dose PE and elevated heavy metal treatments markedly induced increases in malondialdehyde content (MDA) and enhanced the activities of superoxide dismutase (SOD), catalase (CAT), and peroxidase (POD). Under co-contaminated conditions, the addition of low-dose PE reduced the translocation capacity of heavy metals, alleviated heavy metal-induced oxidative stress responses, and promoted tobacco growth. Conversely, high-dose PE promoted the translocation of Cd into tobacco plants and increased Cd contents in tobacco leaves, leading to marked decreases in soluble protein and soluble sugar contents, and causing severe reductions in plant height, number of functional leaves, and biomass. Structural equation modeling (SEM) analysis revealed that the direct effect of PE on tobacco growth was not significant; instead, it primarily acted as a regulatory factor, exerting either promotional or inhibitory effects on tobacco growth at different doses. The impact of Cd/Pb on tobacco growth appeared to involve two potential pathways. On the one hand, Cd/Pb induced direct toxicity through their accumulation within tobacco tissues. On the other hand, they exerted indirect regulation primarily by modulating the activities of the tobacco antioxidant system.

## 1. Introduction

Microplastics (MPs) are defined as plastic fragments with a particle size smaller than 5 mm [1]. As an emerging contaminant, MPs have been widely detected across aquatic and terrestrial environments worldwide [2,3]. Among these, the abundance of MPs in terrestrial systems far exceeds that in aquatic environments [4]. Terrestrial soils act as both a major source and sink for MPs [5]. As the primary substrate for food production, microplastic pollution in agricultural soils and its implications for food security have become a focal topic of current research. Studies have shown that MPs possess a high specific surface area and abundant hydrophilic functional groups [6]. Long-term accumulation of MPs in soils may alter soil physicochemical properties, affect soil aeration and water-holding capacity, and influence plant nutrient uptake [7,8].

Cadmium (Cd) and lead (Pb) are common heavy metal contaminants in soil environments. Owing to their high toxicity and strong bioaccumulation potential, they have become a major public concern [9,10]. Once released into soils, Cd and Pb can be taken up by plant roots and accumulate in crop tissues, disrupting normal physiological and metabolic processes and resulting in growth inhibition, oxidative damage, and yield reduction [11]. For example, a previous study reported that biomass of tobacco and potato grown in Cd- and Pb-contaminated soils decreased significantly, accompanied by pronounced DNA damage. Heavy metals readily accumulate in various plant organs, including roots, stems, leaves, and flowers, thereby interfering with normal growth and metabolism. Such interference may include the suppression of photosynthesis, disruption of cell membrane integrity, and induction of oxidative stress responses, ultimately leading to growth inhibition, reduced yield, and even plant death [12].

Studies have indicated that MPs can act as carriers of contaminants, altering the chemical speciation of heavy metals and thereby affecting plant growth and physiological metabolism [13,14]. Under high pollution levels, the presence of MPs may enhance the mobility of heavy metals, leading to increased uptake by plants and exacerbating toxic effects [15,16]. MPs may influence heavy metal mobility through adsorption and complexation, thereby changing their distribution forms in soils and, consequently, affecting plant uptake and the degree of toxicity [17,18]. Guo et al. reported that MPs can mask soil reactive sites associated with oxygen-containing functional groups and soil inorganic salts (e.g., carbonates, reduced phosphorus species, and hydroxides), thereby reducing the chemisorption of Cd. This, in turn, increases Cd desorption and bioavailability in soils, making it more reactive and toxic [19].

When the bioavailability of heavy metals is altered by MPs and subsequently absorbed by plant roots, the resulting elevation in metal concentrations in plant tissues triggers a cascade of physiological and biochemical defence responses, among which the generation of reactive oxygen species (ROS) and the activation of the antioxidant defence system play a central role in mediating plant tolerance to such combined stress [20]. Reactive oxygen species (ROS) are oxygen metabolites accumulated by plants under environmental stress, mainly including superoxide anion ($O_{2}^{\cdot -}$), hydrogen peroxide ($H_{2}O_{2}$), and hydroxyl radical (·OH) [21]. Elevated ROS levels in plants often serve as a crucial signal of cellular oxidative stress. This can be characterized by the content of malondialdehyde (MDA), a product of membrane lipid peroxidation, along with the activities of antioxidant enzymes such as superoxide dismutase (SOD), peroxidase (POD), and catalase (CAT), thereby evaluating the degree of stress response and tolerance of plants to environmental stress [21,22]. Under co-contamination, heavy metals (such as Pb and Cd) can induce ROS accumulation in plants and disrupt their antioxidant enzyme activities [23]. Meanwhile, microplastics may exacerbate the imbalance of the antioxidant system by altering the bioavailability of contaminants, thereby posing more complex combined stress effects on plant health [24]. Some studies have reported that high concentrations of microplastics can promote the translocation of heavy metals into plants, leading to excessive ROS accumulation, which subsequently triggers an intense response of the antioxidant enzymes [25]. For instance, under the 0.1% PE and Cu/Pb combined treatment, the activities of SOD and POD, as well as the MDA content in rapeseed plants, all increased significantly, exhibiting a pronounced ROS response [26]. Pinto-Poblete et al. [27] found that the treatment with 0.02% PE and 3 mg/kg Cd significantly enhanced the accumulation of Cd in strawberries and significantly affected their yield and biomass. In contrast, another study found that the addition of PLA and PE to Cd-contaminated soils could reduce Cd accumulation in Chinese cabbage and alleviate the growth stress induced by heavy metals [28]. Additionally, research on the effects of combined PS microplastics and Cd contamination on maize growth revealed that the addition of PS microplastics could, to a certain extent, alleviate the growth toxicity of Cd to maize seedlings [29]. In summary, there is currently no unified consensus regarding the mechanisms by which microplastic-heavy metal co-contamination affects the plant antioxidant system. This discrepancy may stem from the complex interactive effects of multiple factors, including the types and concentrations of contaminants, soil types, and plant species and physiological characteristics [17,30].

Due to mineral resource exploitation in the Southwestern region of China, including mining, tailings stockpiling, and ore processing, combined with residual plastic mulching in farmlands, agricultural soils commonly exhibit excessive heavy metal levels and microplastic contamination [7,31]. To date, studies have examined the impacts of microplastic-heavy metal co-contamination on several crops, including Chinese cabbage [32], lettuce [33,34], rapeseed [26], wheat [35,36], sorghum [37], rice [38,39,40] and maize [29]. However, there are few reports concerning its effects on tobacco (*Nicotiana tabacum* L.), an important cash crop, particularly with respect to growth and antioxidant defense. Therefore, using tobacco as the model species, this study employed pot experiments with graded contamination treatments to investigate the effects of PE MPs and Cd/Pb co-contamination on tobacco growth and antioxidant systems, thereby providing a scientific basis for risk assessment and remediation of related environmental pollutants.

## 2. Results

### 2.1. Tobacco Growth Indicators

**Figure 1:**
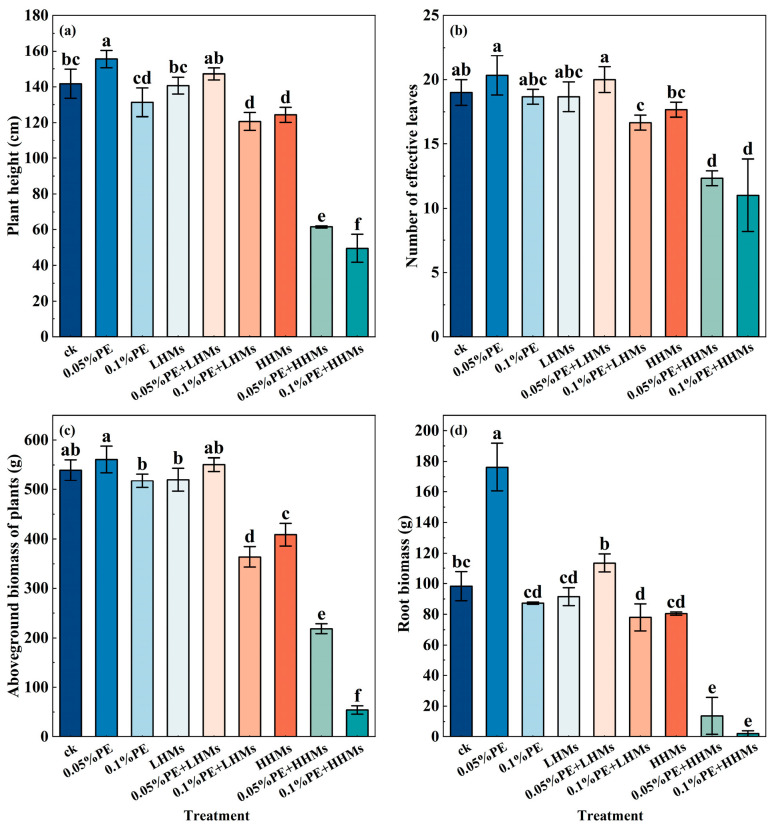
Growth parameters of tobacco: (**a**) plant height, (**b**) number of effective leaves, (**c**) aboveground biomass, and (**d**) underground biomass. Data are presented as mean ± standard deviation (mean ± SD, n = 3). Differences among treatments were analyzed using one-way ANOVA followed by Duncan’s multiple range test. Different lowercase letters indicate statistically significant differences among treatments (*p* < 0.05).

Significant differences were observed in growth parameters, including plant height, number of effective leaves, and biomass, among the different treatments (Figure 1). Compared with CK, slight increases were recorded under 0.05% PE and 0.05% PE + LHMs, with root biomass under 0.05% PE increasing by 77.5%. In contrast, 0.1% PE, LHMs, 0.1% PE + LHMs, and HHMs all reduced growth indicators to varying degrees. The most pronounced inhibition occurred under 0.1% PE + HHMs, with shoot biomass reduced by 90.1% relative to CK.

### 2.2. Soluble Compounds

**Figure 2:**
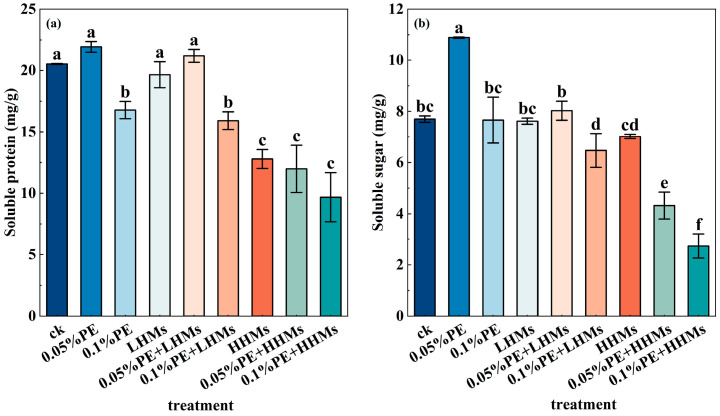
Soluble substance contents in tobacco leaves: (**a**) soluble protein content and (**b**) soluble sugar content. Data are presented as mean ± standard deviation (mean ± SD, n = 3). Differences among treatments were analyzed using one-way ANOVA followed by Duncan’s multiple range test. Different lowercase letters indicate statistically significant differences among treatments (*p* < 0.05).

Different treatments exerted significant effects on the contents of soluble protein and soluble sugars in tobacco (Figure 2). Compared with CK, both 0.05% PE and 0.05% PE + LHMs increased soluble protein and soluble sugar levels, with the most pronounced increase observed under 0.05% PE, where soluble sugar content rose by 41.6% relative to CK. In contrast, 0.1% PE, LHMs, 0.1% PE + LHMs, and HHMs caused decreases of varying degrees in both classes of soluble compounds, with the strongest reductions occurring under 0.05% PE + HHMs and 0.1% PE + HHMs. Under 0.1% PE + HHMs, soluble protein and soluble sugar contents decreased by approximately 52.7% and 65.0% compared with CK, respectively.

### 2.3. Heavy Metal Concentrations in Tobacco Plants

**Figure 3:**
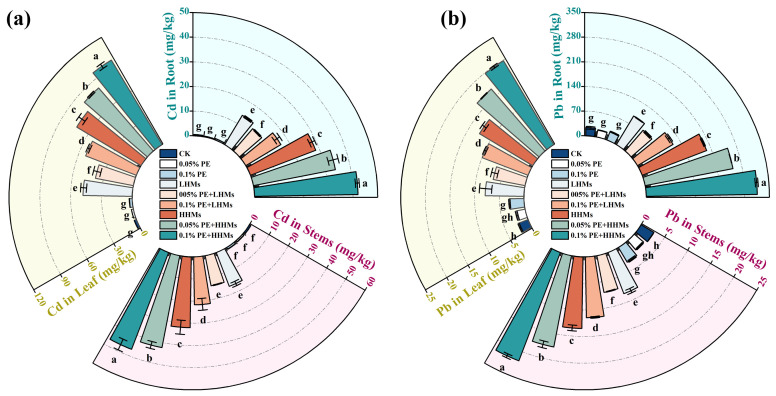
Heavy metal contents in different parts of tobacco: (**a**) Cd and (**b**) Pb. Data are presented as mean ± standard deviation (mean ± SD, n = 3). Differences among plant parts were analyzed using one-way ANOVA followed by Duncan’s multiple range test. Different lowercase letters indicate statistically significant differences (*p* < 0.05).

Marked differences were observed in the tissue-specific accumulation of Cd and Pb in tobacco (Figure 3). Cadmium was predominantly enriched in leaves, exhibiting the trend of leaf > stem > root. In contrast, lead accumulated mainly in roots, following the order root > stem > leaf. Significant differences in heavy metal concentrations were also detected among treatments. Heavy metal concentrations in various tissues of the tobacco cultivar used in this study (cv. Yunyan 87) have been reported to be significantly higher than those of other tobacco varieties, which is consistent with the findings of the present study [41,42]. Under microplastic-only treatments (0.05% PE, 0.1% PE), Cd and Pb concentrations in plant tissues did not differ significantly from CK. Compared with heavy metal-only treatments (LHMs, HHMs), the combined treatments with MPs resulted in significantly higher heavy metal concentrations in plant tissues (*p* < 0.05). Under LHMs conditions, the addition of 0.05% PE showed an antagonistic effect, resulting in slightly lower tissue heavy metal levels compared with LHMs alone. However, increasing PE dose to 0.1% elevated leaf Cd content by approximately 10.4% over LHMs alone. Under HHMs conditions, 0.1% PE + HHMs produced the most pronounced accumulation, with leaf Cd reaching 96.2 mg/kg (a 31.9% increase over HHMs alone at 72.9 mg/kg) and root Pb peaking at 315.4 mg/kg.

### 2.4. Antioxidant Enzyme System

**Figure 4:**
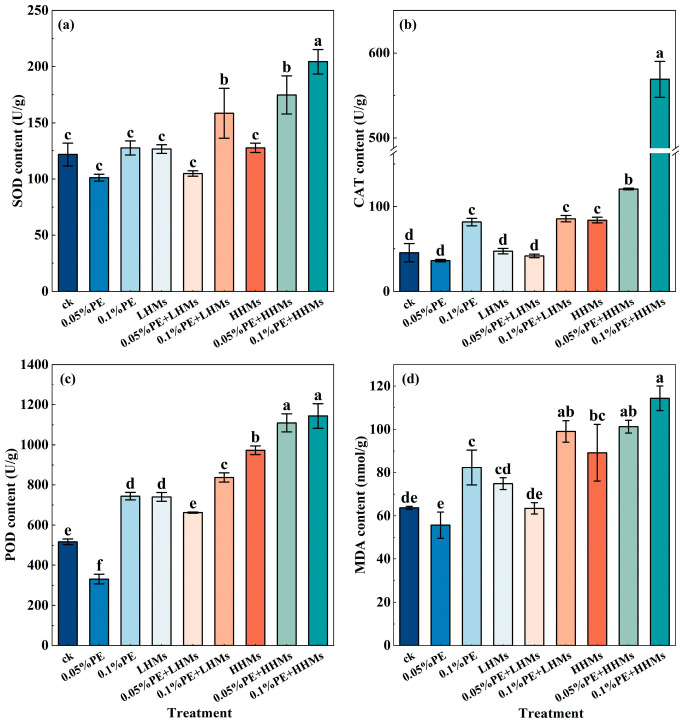
Variations in the antioxidant enzyme system of tobacco leaves: (**a**) SOD, (**b**) CAT, (**c**) POD, and (**d**) MDA. Data are presented as mean ± standard deviation (mean ± SD, n = 3). Differences among treatments were analyzed using one-way ANOVA followed by Duncan’s multiple range test. Different lowercase letters indicate statistically significant differences (*p* < 0.05).

Significant differences were observed in the activities of antioxidant enzymes CAT, POD, and SOD, as well as in MDA content, in tobacco leaves among the different treatments (Figure 4). Relative to CK and LHMs, the 0.05% PE and 0.05% PE + LHMs treatments showed lower antioxidant enzyme activities and MDA levels. By contrast, 0.1% PE, LHMs, 0.1% PE + LHMs, and HHMs exhibited increasing trends, with the most pronounced increases under 0.05% PE + HHMs and 0.1% PE + HHMs. Notably, the 0.1% PE + HHMs treatment yielded the highest antioxidant enzyme activities and MDA content (Figure 4).

## 3. Discussion

### 3.1. Effects of Microplastics on Cd/Pb Accumulation and Translocation in Tobacco

The accumulation sites of the heavy metals Cd and Pb in tobacco exhibited significant differences. In the present study, Cd primarily accumulated in the leaves, followed by the roots and stems, whereas Pb was mainly concentrated in the roots, followed by the stems and leaves (Figure 3). The bioconcentration factor (BF = 1.44–13.13) and translocation factor (TF = 1.00–5.06) values of Cd in various parts of the tobacco plants were significantly higher than those of Pb (BF = 0.01–0.4, TF = 0.06–0.95). This indicates that Cd has stronger accumulation and translocation capacities within tobacco plants, which is likely associated with the selective expression of metal transporter genes (such as NRAMP, ZIP, and HMA) that facilitate the long-distance transport of Cd. At the molecular level, recent studies have confirmed at the transcriptional level (RT-qPCR) that Cd stress significantly upregulates specific metal transporter genes (e.g., N6b-Q) in tobacco, thereby enhancing its systemic translocation [43]. The observed TF values (1.00–5.06) in present work are consistent with these established molecular mechanisms in tobacco [44,45]. The relatively low BF and TF values of Pb suggest that the upward translocation capacity of Pb from the roots is limited. This could be attributed to the strong adsorption of Pb by plant roots, as well as the restriction of its translocation to the aboveground parts through intracellular sequestration and binding ligands (e.g., phytochelatins and cytosolic binding proteins) by the plants [20,46]. Even under the CK treatment without exogenous Cd addition, tobacco still exhibited a Primary TF of 1.56 and a Total TF of 4.39 at a background soil Cd level of only 0.29 mg/kg, indicating that tobacco possesses an inherently strong root-to-leaf Cd translocation capacity that operates effectively even at low soil Cd concentrations. This observation carries direct food and inhalation safety implications, as it suggests that tobacco grown on soils with only moderate Cd contamination may still accumulate substantial Cd in its leaves, posing potential health risks through cigarette consumption. This further underscores the necessity of strict soil Cd screening and risk assessment for tobacco cultivation areas, even where soil Cd levels appear well below conventional contamination thresholds.

Under the treatments with PE microplastics alone (0.05% and 0.1%), the BF and TF values of Cd and Pb in tobacco showed no significant differences compared with the CK group, indicating that in the absence of exogenous heavy metal stress, the impact of microplastics on the translocation of heavy metals in the soil–tobacco system is relatively limited (Table 1). Under the addition of low concentrations of heavy metals (LHMs), the BF and TF of Cd and Pb in tobacco were not significantly different from those in the CK, suggesting that at low heavy metal pollution levels, the capacity of heavy metals to translocate to the aboveground parts is also limited. However, the BF and TF of Cd in tobacco under the 0.05% PE + LHMs treatment decreased compared to the LHMs treatment alone. This might be influenced by the competitive adsorption of heavy metals by microplastics, which leads to a decrease in the proportion of available heavy metals in the soil, thereby reducing heavy metal accumulation in plants [28,47]. Conversely, the BF and TF values of Cd and Pb in tobacco leaves under the 0.1% PE + LHMs treatment were both higher than those under the LHMs treatment, indicating that high doses of microplastics may promote the translocation and plant uptake of Cd and Pb in the soil. Previous studies have found that microplastics at doses of 1% and 10% increased the desorption of Cd in the soil by altering soil pH, organic matter content, and aggregate structure, thereby enhancing the mobility and bioavailability of heavy metals and leading to the uptake of more Cd ions by plants [47]. Similar conclusions were also drawn in a rapeseed experiment, where the addition of 0.1% PE microplastics to the soil significantly increased the translocation capacity of Cu and Pb, and promoted their accumulation in rapeseed plants [26]. In contrast to these literature reports, although PE microplastics have been reported to lower soil pH and thereby enhance Cd solubility in some studies, rhizosphere pH measurements in this study (Supplementary Materials Table S1) showed no significant differences across treatments (pH range: 4.32–4.47, *p* > 0.05). This indicates that pH-mediated changes in Cd solubility are unlikely to be the primary driver of the enhanced Cd accumulation observed under high-dose PE treatments. In addition to these abiotic soil factors, PE microplastics may also alter rhizosphere microbial communities and arbuscular mycorrhizal fungi (AMF) symbiosis, which are recognised as key regulators of heavy metal bioavailability and root uptake under abiotic stress conditions through strigolactone-mediated signalling pathways [48].

Under the high concentrations of heavy metal (HHMs) treatments, the BF and TF values of Cd and Pb in tobacco decreased slightly compared with those under the LHMs treatments. However, the BF and TF values of Cd and Pb in tobacco under the 0.05% PE + HHMs and 0.1% PE + HHMs treatments were significantly higher than those in other treatments (*p* > 0.05), indicating that under a high heavy metal background, the addition of either high-dose or low-dose microplastics can enhance the translocation and accumulation capacities of heavy metals in plants. Notably, the Cd accumulation in tobacco leaves under the high-dose combined treatment (0.1% PE + HHMs) in this study was extremely high (Cd = 96.0 mg/kg, BF = 9.37), which was significantly higher than that in other crops under the same pollution load. This extraordinary Cd accumulation phenomenon might be closely related to the specific physiological characteristics of tobacco for high Cd accumulation. For example, Guo et al. (2024) found that under a high Cd background (Cd = 0.18–3.81 mg/kg), the average BF value of Cd in farmland tobacco leaves was 8.02, and could reach up to 38.56 in extreme cases [49]. This study not only artificially added exogenous Cd (5–10 mg/kg) but also superimposed the strong synergistic activation effect of microplastics on soil heavy metals.

### 3.2. Effects of Microplastic and Cd/Pb Co-Contamination on the Growth and Antioxidant System of Tobacco

High levels of heavy metal accumulation in plants often trigger a series of oxidative stress responses and affect plant growth and development [50]. The results showed that the high accumulation levels of Cd and Pb in tobacco leaves were closely related to the changes in antioxidant enzyme activities (such as SOD, POD, and CAT) as well as membrane lipid peroxidation (MDA). The growth and physiological responses of tobacco under different treatments exhibited distinct dose-effect characteristics (Figure 4a).

In the present study, the 0.05% PE treatment promoted tobacco growth and development, with plant height, number of effective leaves, and biomass all outperforming the CK. Activities of antioxidant enzymes such as SOD, POD, and CAT, as well as MDA content, were lower than those of the CK, suggesting that at low microplastic concentrations, tobacco may exhibit a favorable antioxidant physiological response, effectively maintaining low lipid peroxidation levels [51,52]. Concurrently, soluble sugar and soluble protein contents showed an increasing trend under 0.05% PE treatment, with soluble sugar increasing by 41.6% compared with CK. It likely represents an early osmotic adjustment signalling response triggered by mild perturbations in ionic and water homeostasis induced by low-dose PE. Under such mild stress conditions, plants typically activate the coordinated accumulation of compatible osmolytes, including soluble sugars and proteins, to stabilise cellular osmotic potential, protect membrane integrity, and supply carbon skeletons for adaptive metabolism [53,54].

By contrast, the 0.1% PE treatment significantly inhibited plant growth. Antioxidant enzyme activities (CAT, POD) and MDA content showed moderate elevations relative to CK, while SOD remained largely unchanged. It is worth noting that recent studies have proposed that MDA, traditionally regarded as an indicator of membrane lipid peroxidation, may also function as an adaptive signalling molecule under mild stress conditions rather than purely a marker of oxidative injury. Based on this framework, The moderate MDA elevation observed under 0.1% PE, accompanied by coordinated upregulation of CAT and POD, is more consistent with an adaptive signalling response that activates downstream antioxidant defence than with overt cellular damage [42]. This dual nature of MDA—serving as a signalling intermediate at low to moderate stress intensities and as a damage marker at high stress intensities—should be considered when interpreting oxidative responses across the dose-response spectrum observed in this study. However, when stress intensified further under high heavy metal background, tobacco was unable to sustain an effective antioxidative defence, leading to cellular injury and metabolic dysfunctions [55]. The decreases in soluble sugar and protein further indicate impaired osmotic regulation and reduced biosynthetic capacity [56], consistent with the observed growth suppression. The stress exerted by high-dose MPs on tobacco growth likely involves multiple factors, including alterations in rhizosphere soil physicochemical properties and the release of toxic substances from the MPs themselves [57]. For example, under high microplastic concentrations (1%), soil bulk density increases significantly relative to the control, total porosity declines, and soil water-holding capacity decreases, thereby affecting nutrient availability and root uptake of water and nutrients, with consequent impacts on plant growth [58]. Moreover, the addition of 0.1% polystyrene MPs rich in methylene and benzene rings, polyethylene MPs, or polypropylene MPs significantly reduced lettuce biomass, markedly increased antioxidant enzyme indices, and significantly decreased soluble substance contents [55].

Under low-dose heavy metal treatment (LHMs), plants exhibited only mild growth inhibition relative to the control (CK), and several physiological and biochemical indicators (e.g., SOD and CAT activities, soluble sugar content) showed limited variation. This suggests that at low contamination levels, plants retain a degree of self-regulation and stress tolerance [59]. By contrast, under high concentrations of Cd/Pb (HHMs), leaf heavy metal accumulation increased, markedly suppressing tobacco growth beyond its physiological tolerance. This likely arises because elevated Cd/Pb induces oxidative stress, disrupts cell membrane integrity, and interferes with normal metabolic pathways [60]. In a related study on pak choi, Li et al. (2025) [61] reported that at a low dose (5 mg/kg), Cd promoted growth via the ROS-IAA-sugar metabolism signaling pathway, whereas a high dose (40 mg/kg) Cd caused severe growth inhibition through ROS-accumulation-mediated hormonal and sugar metabolic dysregulation. Notably, under the combined 0.05% PE + LHMs treatment, tobacco showed improved growth metrics compared with LHMs alone, along with reduced SOD, CAT, and POD activities and lower MDA content. This pattern represents a classic hormesis effect, in which low-dose stressor exposure elicits stimulatory rather than inhibitory effects. At low heavy metal concentrations, the introduction of a low PE dose may have modulated rhizosphere properties in a manner that slightly reduced Cd and Pb bioavailability, thereby relieving heavy metal-induced oxidative burden and allowing the antioxidant system to return toward homeostasis. This dose-dependent biphasic response, where low-dose PE alleviates and high-dose PE exacerbates heavy metal stress, has been reported in tobacco under Cd stress, where plant defence mechanisms are activated in a concentration-dependent manner [17,43].

Under the combined 0.05% PE + HHMs and 0.1% PE + HHMs treatments, tobacco growth and development were severely inhibited: plant height, leaf number, and biomass decreased markedly. Notably, even at the lower PE dose (0.05% PE + HHMs), growth inhibition was more pronounced than under HHMs alone, suggesting that the addition of PE, regardless of dose, exacerbated rather than buffered heavy metal stress on tobacco growth under high metal contamination background. This dose-dependent aggravation was further intensified under 0.1% PE + HHMs, where the most severe reductions in growth parameters were observed, while activities of antioxidant enzymes (SOD, CAT, POD) and MDA content generally increased, which is indicative of a physiological response to intensified oxidative stress and exacerbated membrane damage. Particularly in the 0.1% PE + HHMs group, antioxidant enzyme activities were significantly higher than in other treatments, potentially reflecting a compensatory response to the high heavy metal burdens and extreme stress intensity in leaves. Meanwhile, contents of osmotic adjustment substances such as soluble sugars and soluble proteins declined significantly, pointing to constrained metabolic regulation and broadly impaired physiological activity [62,63]. These findings suggest a pronounced synergistic stress effect between high concentrations of PE MPs and heavy metals [64]. On the one hand, high-dose heavy metals impose severe stress that disrupts normal growth and metabolic homeostasis; on the other, PE MPs further potentiate heavy metal toxicity. At high microplastic levels (1%), PE may directly perturb the peanut rhizosphere environment by forming a physical barrier, thereby reducing root uptake of water and nutrients and further aggravating heavy metal stress on plant growth [65].

### 3.3. Key Pathways and Effects of Combined Microplastic and Heavy Metal Pollution on Tobacco Growth and Antioxidant Systems

**Figure 5:**
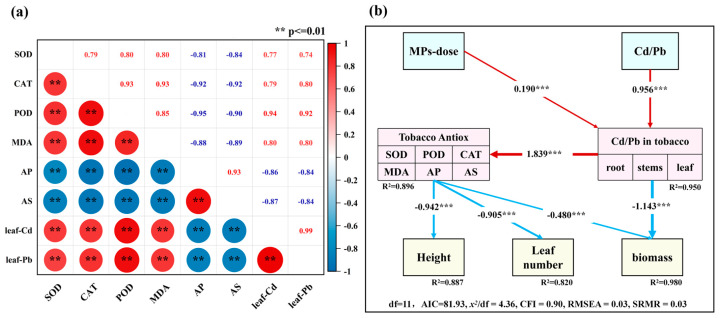
(**a**) Pearson correlation coefficients among various parameters in tobacco leaves, including SOD, CAT, POD, MDA, soluble protein, soluble sugar, Cd in leaf, and Pb in leaf. Color intensity represents the strength of the correlation, with red indicating positive correlations and blue indicating negative correlations. Significance levels are denoted by asterisks: ** *p* < 0.01. (**b**) Structural equation modeling (SEM) of the effects of microplastics and Cd/Pb co-contamination on the antioxidant system and growth and development of tobacco. The thickness of the arrows reflects the strength of the effects, and the numbers represent the standardized path coefficients (β). Significance levels are indicated by asterisks: *** *p* < 0.001.

Correlation analysis was performed among SOD, CAT, POD, MDA, soluble protein, soluble sugar, and heavy metal contents in tobacco leaves (Figure 5a). The results showed that soluble substances were significantly negatively correlated with leaf heavy metal contents and antioxidant system indicators (SOD, CAT, POD, MDA) (all r values < −0.80), whereas antioxidant enzyme indicators were significantly positively correlated with heavy metal concentrations in leaves (all r values > 0.70). Significant positive correlations were also observed among SOD, CAT, POD, and MDA, with the correlation between CAT and POD being the most significant (r = 0.93), indicating that antioxidant system indicators exhibit a certain degree of coordination when coping with environmental stress [56,66]. To further elucidate the effects of microplastics, heavy metals, and the antioxidant system on tobacco growth, a structural equation model (SEM) was constructed. In this model, the doses of microplastics and Cd/Pb were set as exogenous variables; the heavy metal load in tobacco (Cd/Pb in tobacco) and the antioxidant system (Antiox) as mediating variables; and tobacco growth indicators (plant height, number of effective leaves, and biomass) as outcome variables. Structural equation modeling (SEM) was employed to conduct a path analysis of the direct and indirect relationships among the various element variables in the soil–tobacco system (Figure 5b).

The results showed that MPs and Cd/Pb had significant positive effects on the accumulation of heavy metals in tobacco (β = 0.190, β = 0.956, *p* < 0.001). The direct effects of MPs on the tobacco antioxidant system and growth indicators were not significant (*p* > 0.05). The heavy metal load in the plants exhibited a significant direct negative effect on tobacco biomass (β = −1.143, *p* < 0.01), indicating that the accumulation of Cd and Pb in tobacco is a key factor affecting tobacco growth. Meanwhile, heavy metals also indirectly affected the activities of the antioxidant system by enhancing the level of oxidative stress (β = 1.839, *p* < 0.05), thereby further inhibiting tobacco growth. The antioxidant system indicators played a typical “mediating” role in the SEM. They exerted significant negative effect on tobacco plant height, number of effective leaves, and biomass (β = −0.942, β = −0.480, β = −0.905, *p* < 0.001). Combined with the strong positive correlation between heavy metal load and antioxidant system activity, these results define the main impact pathway of “metal load → oxidative stress → tobacco growth”. Although the direct path effect of the microplastic dose on tobacco growth was not significant, it exerted an indirect effect on tobacco growth by altering the translocation capacity of heavy metals and the activities of the antioxidant system. The low-dose (0.05% PE) treatment may reduce the translocation capacity of Cd and Pb by altering soil physicochemical properties, thereby alleviating heavy metal-induced oxidative stress, maintaining the dynamic balance of the antioxidant system, and indirectly promoting tobacco growth. Conversely, the high-dose (0.1% PE) treatment may enhance the bioavailability of heavy metals in the soil, further increase their accumulation in tobacco plants, and exacerbate the oxidative stress and membrane lipid peroxidation responses within tobacco, thereby inhibiting tobacco growth performance.

## 4. Materials and Methods

### 4.1. Microplastics, Heavy Metals, and Soil

The low-density polyethylene (LDPE) microplastics used in this study, with an average particle size of 100 μm, were purchased from Dongguan Zhangmutou Co., Ltd. (Dongguan, China). The characterization of the microplastics was performed using Fourier transform infrared spectroscopy (FTIR) and scanning electron microscopy (SEM), and the results are provided in Figures S1 and S2. For the heavy metal treatments, analytical-grade cadmium chloride (CdCl_2_, molecular weight = 183.32, purity = 99%) and lead chloride (PbCl_2_, molecular weight = 278.11, purity = 99.5%) were utilized as the sources. The selected target concentrations (5 and 10 mg/kg for Cd, and 500 and 1000 mg/kg for Pb) correspond to the typical heavy metal contamination levels found in agricultural soils in China [67,68]. To achieve these target pure metal concentrations, the application rates were strictly calculated based on 20 kg of dry soil per pot. Specifically, to reach the 5 and 10 mg/kg Cd levels, 0.1649 g and 0.3297 g of CdCl_2_ were accurately weighed for each pot, respectively. To reach the 500 and 1000 mg/kg Pb levels, 13.4957 g and 26.9914 g of PbCl_2_ were weighed for each pot, respectively. To ensure quantitative transfer and avoid dry powder loss during application, the precisely weighed salts for each pot were transferred into 50 mL centrifuge tubes containing deionized water. Because of the low solubility of PbCl_2_, it formed an aqueous suspension. The 50 mL Cd and Pb suspensions were then gradually poured into the respective pots to ensure accurate dosing.

The soil used in this study was collected from Maoyun Township, Kaiyang County, Guiyang City, Guizhou Province, China (107.163720° E, 26.970014° N), at a depth of 0~30 cm from tobacco fields. The soil texture is brown clay loam, consisting of 20.90% sand (2.0–0.05 mm), 40.88% silt (0.05–0.002 mm), and 38.22% clay (<0.002 mm). After natural air-drying, the soil was passed through a 10-mesh sieve, and the sieved air-dried soil was used for subsequent experiments. The soil type was Yellow Soil (Huangtu). The corresponding physicochemical properties were as follows: pH = 5.1; cation exchange capacity (CEC) = 4.1 cmol(+)/kg; soil organic carbon (SOC) = 27.9 g/kg; total nitrogen (TN) = 1.29 g/kg; total phosphorus (TP) = 0.407 g/kg; total potassium (TK) = 7.2 g/kg; available phosphorus (AP) = 26.8 mg/kg; available potassium (AK) = 228 mg/kg; alkaline hydrolysable nitrogen (HN) = 135 mg/kg; total Pb = 45.5 mg/kg; available Pb = 7.83 mg/kg; total Cd = 0.29 mg/kg; and available Cd = 0.02 mg/kg.

### 4.2. Experimental Design and Sample Collection

The pot experiment was conducted in a greenhouse. Each pot was filled with 20 kg of air-dried soil. Previous studies have indicated that the microplastic content in Chinese agricultural soils can reach up to 816.57 mg/kg [69], particularly in intensive farming areas or agricultural hotspots with long-term plastic mulching. Considering that microplastic contamination is a continuous and cumulative process, PE concentrations of 0.05% and 0.1% (*w*/*w*) were selected as ecologically relevant levels in this study. These concentrations represent a worst-case scenario for long-term mulch film residue accumulation, which is essential for assessing the potential high-end ecological risks of combined microplastic and heavy metal pollution on tobacco growth and quality. One control (CK) and eight treatment groups were established, with three replicates per group. The experimental treatment design is presented in Table 2. The experiment lasted for 90 days, covering the entire growth cycle of tobacco from transplanting, through the vigorous growth stage, to physiological maturity.

Prior to transplanting, the required amounts of LDPE microplastics and heavy metal solutions were added to each pot simultaneously and thoroughly mixed into the soil. The soils amended with microplastics and heavy metals were thoroughly mixed and aged at room temperature for 10 days to allow for the initial equilibration of heavy metal speciation and their preliminary interaction with the soil matrix and microplastics. Subsequently, one tobacco seedling (cv. Yunyan 87) at the five-leaf stage with uniform growth status was transplanted into each pot. To simulate the nutrient supply per plant in local agronomic practices, a compound fertilizer (136.37 g per pot; 9% N, 10% $P_{2}O_{5}$, and 27% $K_{2}O$, supplied by Guizhou Ketai Jinfu Fertilizer Co., Ltd., Fuquan, China) was applied as the basal fertilizer. This application rate was calculated by converting the local field basal fertilization rate (a mixture of inorganic and organic fertilizers, approximately 205 kg per mu at a planting density of 1100 plants per mu) into the equivalent amount of pure compound fertilizer per seedling. Although this application rate results in a higher soil ionic strength in a closed pot compared to open field conditions, the use of a large soil volume (20 kg per pot) provided sufficient buffering capacity to avoid phytotoxicity. Furthermore, this basal application was uniform across all treatments to ensure a consistent background level for soil chemistry and metal availability. Topdressing was then performed at 35 and 50 days after transplanting, respectively. The greenhouse was maintained at a temperature of 20–30 °C and a relative humidity of approximately 60%, under a 16 h light/8 h dark photoperiod. During the cultivation period, the positions of the pots were randomly rotated every 3 days to ensure uniform light exposure, and the pots were watered regularly to maintain optimal soil moisture. Local tap water complying with the Chinese national drinking water quality standard (GB 5749-2022) was used uniformly for irrigation across all treatments, with Cd and Pb concentrations well below the regulatory limits (Cd ≤ 0.005 mg/L; Pb ≤ 0.01 mg/L), which were negligible compared with the exogenously added heavy metal concentrations in this study. At 90 days post-transplanting, plant height and number of effective leaves were recorded. From each plant, a mature and healthy middle-position leaf was harvested, rinsed, air-dried, flash-frozen in liquid nitrogen, and stored in centrifuge tubes at −80 °C for biochemical analyses.

### 4.3. Plant Height, Leaf Number, and Biomass Determination

For each tobacco plant, plant height was measured, and number of effective leaves was recorded. Harvested plants were separated into belowground parts (roots) and aboveground parts (stems and leaves), rinsed with deionized water, and oven-dried at 105 °C to constant weight.

### 4.4. Determination of Biochemical Indices in Tobacco Leaves

To quantify peroxidase (POD), superoxide dismutase (SOD), catalase (CAT), soluble protein, and malondialdehyde (MDA), 0.1 g of tobacco leaf tissue was ground to a fine powder in liquid nitrogen and homogenized in 1 mL phosphate-buffered saline (PBS, Solarbio, Beijing, China). The homogenate was centrifuged at 4 °C and 8500 rpm for 10 min, and the supernatant was collected. Following the method of Lian et al. [70], absorbance was measured using a UV-Vis spectrophotometer (KBT2103011, Unico, Shanghai, China) at the corresponding wavelengths: SOD at 560 nm, POD at 470 nm, CAT at 240 nm, soluble protein at 540 nm, and MDA at 532 and 600 nm. For soluble sugar determination, 0.1 g of leaf tissue was extracted with 1.0 mL distilled water in a 95 °C water bath for 10 min. After cooling, the extract was centrifuged at 8500 rpm for 10 min, and the supernatant was analyzed using the anthrone colorimetric method at 620 nm to determine soluble sugar content. The activities of SOD, POD, and CAT were expressed in U/g, and the content of MDA was expressed in nmol/g, all calculated on a fresh weight basis.

### 4.5. Heavy Metal Determination

After harvest, tobacco leaf samples were oven-dried to constant weight and ground to pass a 100-mesh sieve. An aliquot of 0.2 g was weighed into a glass digestion vessel, followed by the addition of 8 mL ${HNO}_{3}$ and 2 mL ${HCIO}_{4}$, and the sample was left to predigest overnight at room temperature. The vessel was then placed on a hot plate and heated progressively: first at 120 °C until brown fumes had completely dissipated, then raised to 180–200 °C and maintained until the solution became clear and was reduced to approximately 0.5–1 mL. After cooling, the digest was brought to a final volume of 25 mL with 1% ${HNO}_{3}$. Upon completion, the digest was brought to a final volume of 25 mL. Cadmium (Cd) and lead (Pb) concentrations in the solution were determined by inductively coupled plasma mass spectrometry (ICP-MS, ICAP-RQ, Thermo Fisher Scientific, Waltham, MA, USA). A procedural blank, replicate samples, and the certified reference material (Orange Leaf, GBW-10020, Beijing, China) were processed in parallel with each digestion batch to ensure data quality. The recovery rate of the certified reference material was greater than 95%, confirming the accuracy and reliability of the analytical procedure.

### 4.6. Calculation of Bioaccumulation and Translocation Factors

To evaluate the accumulation and step-wise migration capacity of Cd and Pb in tobacco, the bioaccumulation factor (BF) and translocation factors (TF) were calculated based on the heavy metal concentrations in the rhizosphere soil and the roots, stems, and leaves of the tobacco plants.

(1) Bioaccumulation Factor (BF):

BF = C_plant_/C_soil_

where C_plant_ is the concentration of Cd or Pb in a specific part of the tobacco plant (root, stem, or leaf, mg/kg), and C_soil_ is the total concentration of the corresponding heavy metal in the rhizosphere soil under the specific treatment (mg/kg). A higher BF value indicates a stronger accumulation capacity of the plant for the heavy metal.

(2) Translocation Factors (TF):

TFs were used to characterize the mobility of heavy metals within the plant tissues at different levels:

Primary TF = C_stem_/C_root_

Secondary TF = C_leaf_/C_stem_

Total TF = C_leaf_/C_root_

where C_root_, C_stem_, and C_leaf_ represent the concentrations of Cd or Pb in the roots, stems, and leaves, respectively. Higher TF values indicate a stronger capacity for heavy metals to migrate upward step-by-step or totally towards the functional organs (leaves).

### 4.7. Data Processing and Analysis

All results are expressed as mean ± standard deviation (mean ± SD) based on three replicates. Data processing and statistical analyses were performed using Microsoft Excel 2019 (Microsoft Corp., Redmond, WA, USA) and IBM SPSS Statistics 29.0 (IBM Corp., Armonk, NY, USA), and figures were generated with Origin 2025b (OriginLab, Northampton, MA, USA). Differences between the treatment groups were evaluated using a one-way analysis of variance (ANOVA), followed by Duncan’s multiple range test to determine statistical significance (n = 3, *p* < 0.05). Structural equation modeling (SEM) was further conducted using IBM SPSS Amos 29.0 (IBM Corp., Armonk, NY, USA) to explore the mechanistic pathways among pollutant exposure, metal accumulation, antioxidant responses, and plant growth. Prior to model construction, all variables were standardized (Z-score transformation) in SPSS 29.0. To address the limitation of a small sample size (n = 27) and overcome severe multicollinearity, dimensional reduction was first achieved via principal component analysis (PCA). Specifically, Cd and Pb concentrations in roots, stems, and leaves were integrated into a single metal load component (Cd/Pb in tobacco), while physiological parameters (SOD, POD, CAT, MDA, soluble protein (AP), and soluble sugar (AS)) were consolidated into an antioxidant component (Antiox). This pre-processing drastically reduced the number of free parameters, ensuring the robustness and statistical power of the subsequent structural equation modeling (SEM). Model parameters were then estimated using the maximum likelihood estimation (MLE) method, establishing an initial conceptual path linking “Pollution factor–Metal load–Antioxidant response–Growth traits”. The model was iteratively refined based on significance testing, and the final goodness-of-fit and explanatory power were evaluated using the coefficient of determination (R2) and Akaike information criterion (AIC). The final path diagram was visualized using Adobe Illustrator 2019 (Adobe Inc., San Jose, CA, USA).

## 5. Conclusions

Low-dose PE or low concentrations of Cd/Pb had limited effects on the plants; furthermore, the combined treatment of low-dose PE and low concentrations of Cd/Pb alleviated metal toxicity, promoted tobacco growth, and reduced Cd/Pb accumulation in leaves. In contrast, high-dose PE combined with high Cd/Pb concentrations synergistically intensified oxidative stress, suppressed soluble protein and sugar synthesis, and severely inhibited tobacco growth. There are distinct differences in the translocation patterns of Cd and Pb within the plants: Cd is more prone to translocate to the aboveground parts, whereas Pb is mainly retained in the roots. However, under high-concentration co-contamination, PE further promoted the accumulation of Cd in the aboveground parts. Structural equation modeling (SEM) confirmed that microplastic–heavy metal co-contamination indirectly inhibits tobacco growth by promoting heavy metal accumulation and disrupting the antioxidant defense system. Future studies are warranted to incorporate photosynthetic parameters, rhizosphere microbial community analysis, multi-cultivar comparisons, and a broader range of microplastic particle sizes, in order to more comprehensively elucidate the effects of microplastic–heavy metal co-contamination on tobacco.

## Figures and Tables

**Table 1:** Bioaccumulation factors (BF) and translocation factors (TF) of cadmium and lead in tobacco under different treatments.

**Treat**	**Root BF (Root/Soil)**		**Stem BF (Stem/Soil)**		**Leaf BF (Leaf/Soil)**	
	**Cd BF**	**Pb BF**	**Cd BF**	**Pb BF**	**Cd BF**	**Pb BF**
ck	2.73 ± 0.39 bc	0.40 ± 0.10 a	4.26 ± 0.74 ab	0.05 ± 0.00 a	13.13 ± 0.64 a	0.04 ± 0.01 a
0.05% PE	1.44 ± 0.09 d	0.27 ± 0.01 bcd	2.20 ± 1.92 c	0.03 ± 0.00 b	7.68 ± 0.01 d	0.02 ± 0.00 b
0.1% PE	2.32 ± 0.13 bcd	0.34 ± 0.02 ab	3.85 ± 0.29 ab	0.04 ± 0.00 a	11.71 ± 0.49 b	0.04 ± 0.00 a
LHMs	2.64 ± 0.08 cd	0.16 ± 0.00 ef	3.12 ± 0.20 abc	0.01 ± 0.01 e	9.12 ± 0.56 c	0.01 ± 0.00 de
0.05% PE + LHMs	2.28 ± 0.06 bcd	0.14 ± 0.01 f	2.64 ± 0.03 bc	0.01 ± 0.00 de	7.15 ± 0.69 d	0.01 ± 0.00 f
0.1% PE + LHMs	3.17 ± 0.25 ab	0.20 ± 0.01 def	4.12 ± 0.51 ab	0.02 ± 0.00 c	10.07 ± 0.27 c	0.02 ± 0.00 cd
HHMs	2.77 ± 0.20 bc	0.19 ± 0.01 ef	3.12 ± 0.31 abc	0.01 ± 0.00 de	7.30 ± 0.31 d	0.01 ± 0.00 f
0.05% PE + HHMs	3.30 ± 0.20 ab	0.24 ± 0.00 cde	4.12 ± 0.16 ab	0.02 ± 0.00 cd	7.55 ± 0.66 d	0.02 ± 0.00 cde
0.1% PE + HHMs	4.10 ± 0.08 a	0.29 ± 0.00 bc	4.56 ± 0.26 a	0.02 ± 0.00 c	9.37 ± 0.24 c	0.02 ± 0.00 c
**Treat**	**Primary TF (Stem/Root)**		**Secondary TF (Leaf/Stem)**		**Total TF (Leaf/Root)**	
	**Cd TF**	**Pb TF**	**Cd TF**	**Pb TF**	**Cd TF**	**Pb TF**
ck	1.56 ± 0.09 abc	0.12 ± 0.02 a	2.82 ± 0.04 a	0.75 ± 0.06 c	4.39 ± 0.20 b	0.09 ± 0.01 b
0.05% PE	1.86 ± 0.68 a	0.11 ± 0.01 ab	2.58 ± 0.54 ab	0.81 ± 0.14 ab	5.24 ± 0.37 a	0.09 ± 0.01 b
0.1% PE	1.66 ± 0.10 ab	0.13 ± 0.01 a	3.05 ± 0.18 a	0.92 ± 0.10 a	5.06 ± 0.41 a	0.12 ± 0.01 a
LHMs	1.22 ± 0.07 bc	0.07 ± 0.03 c	2.94 ± 0.26 a	0.87 ± 0.05 ab	3.48 ± 0.18 c	0.09 ± 0.01 b
0.05% PE + LHMs	1.16 ± 0.03 bc	0.09 ± 0.00 bc	2.71 ± 0.23 a	0.94 ± 0.09 a	3.14 ± 0.27 c	0.08 ± 0.01 b
0.1% PE + LHMs	1.31 ± 0.18 bc	0.10 ± 0.00 b	2.47 ± 0.31 abc	0.87 ± 0.03 ab	3.20 ± 0.33 c	0.08 ± 0.00 b
HHMs	1.00 ± 0.01 c	0.07 ± 0.01 c	2.64 ± 0.52 ab	0.94 ± 0.06 a	2.57 ± 0.31 d	0.06 ± 0.00 c
0.05% PE + HHMs	1.25 ± 0.12 bc	0.07 ± 0.00 c	1.93 ± 0.05 c	0.95 ± 0.03 a	2.40 ± 0.17 d	0.07 ± 0.00 c
0.1% PE + HHMs	1.11 ± 0.08 bc	0.07 ± 0.00 c	2.06 ± 0.17 bc	0.91 ± 0.00 ab	2.29 ± 0.01 d	0.06 ± 0.00 c

Notes: BF values were calculated as the ratio of heavy metal concentration in plant tissue to total heavy metal concentration in rhizosphere soil. Values are means of three replicates ± SD. Different lowercase letters indicate significant differences among treatments (*p* < 0.05).

**Table 2:** Test pollutant design.

Treat	PE (mg/kg)	Cd (mg/kg)	Pb (mg/kg)
ck	0	0	0
0.05% PE	500	0	0
0.1% PE	1000	0	0
LHMs	0	5	500
0.05% PE + LHMs	500	5	500
0.1% PE + LHMs	1000	5	500
HHMs	0	10	1000
0.05% PE + HHMs	500	10	1000
0.1% PE + HHMs	1000	10	1000

## Data Availability

The data presented in this study are available upon request from the corresponding author due to privacy and confidentiality restrictions associated with the ongoing research project.

## Supplementary Materials

The following supporting information can be downloaded at: https://www.mdpi.com/article/10.3390/plants15111755/s1, Table S1. Hysicochemical properties of rhizosphere soil under different treatments. Figure S1. Scanning electron microscopy (SEM) micrographs of polyethylene (PE) samples at different magnifications. The scale bars represent (a) 200 μm and (b) 50 μm. Figure S2. FTIR spectrum of the polyethylene (PE) microplastics used in the study.
