# Supplementary material for: Effects of Microplastics and Cd/Pb Co-Contamination on Tobacco (Nicotiana tabacum L.) Growth and Antioxidant Systems
Source: Plants (Basel). 2026 Jun 5;15(11):1755. doi: 10.3390/plants15111755 (PMC13259306; doi:10.3390/plants15111755)
Supplement: Supplementary file 1 [file plants-15-01755-s001.zip › plants-4275734-supplementary.pdf]

Supplementary Materials

Title: Effects of Microplastics and Cd/Pb Co-Contamination on Tobacco (*Nicotiana tabacum* L.) Growth and Antioxidant Systems

Authors: Shengxue Guan [1], Yiwen Hu [1], Ke Jiang [1,2,*], Taoze Liu [1,2], Jiegang Liu [1], Hui Wang [1,2] and Zhanghong Wang [1,2]

[1] College of Eco-Environmental Engineering, Guizhou Minzu University, Guiyang 550025, China

[2] Engineering Research Center of Green and Low-Carbon Technology for Plastic Application, Guizhou Minzu University, Guiyang 550025, China

* Correspondence: jiangke@gzmu.edu.cn

Corresponding author: Ke Jiang

Email: jiangke@gzmu.edu.cn

Table: 1

Figures: 2

Contents

Table S1. Hysicochemical properties of rhizosphere soil under different treatments.

Figure S1. Scanning electron microscopy (SEM) micrographs of polyethylene (PE) samples at different magnifications. The scale bars represent (a) 200 μm and (b) 50 μm.

Figure S2. FTIR spectrum of the polyethylene (PE) microplastics used in the study.

Table S1. Hysicochemical properties of rhizosphere soil under different treatments.

Treatment	pH	CEC<br>Cmol(+).kg^{-1}	SOC<br>g.kg^{-1}	TN<br>g.kg^{-1}	TP<br>g.kg^{-1}	TK<br>g.kg^{-1}	HN<br>mg.kg^{-1}	AP<br>mg.kg^{-1}	AK<br>mg.kg^{-1}
Ck	4.37±0.06^{a}	12.12±0.38^{ab}	26.85±0.65^{abc}	0.15±0.01^{a}	0.83±0.15^{c}	9.85±0.65^{a}	261.00±19.00^{b}	120.60±10.90^{e}	2020.50±96.50^{bc}
0.05%PE	4.38±0.05^{a}	11.94±0.68^{ab}	29.25±1.05^{ab}	0.15±0.02^{a}	0.92±0.04b^{c}	10.1±0.75^{a}	198.00±3.00^{cd}	174.65±5.95^{d}	1905.33±94.11^{d}
0.1%PE	4.38±0.10^{a}	11.59±0.20^{b}	26.73±1.05^{bc}	0.15±0.02^{a}	0.86±0.12^{c}	10.33±0.12^{a}	321.00±36.00^{a}	134.40±0.60^{e}	2196.33±141.68^{ab}
LHMs	4.37±0.14^{a}	11.65±0.16^{b}	27.10±1.73^{abc}	0.15±0.01^{a}	0.98±0.16b^{c}	9.63±0.35^{a}	190.00±39.00^{cd}	235.45±1.25^{c}	1613.00±275.00^{e}
0.05%PE+LHMs	4.33±0.08^{a}	12.14±0.62^{ab}	25.70±1.50^{c}	0.15±0.02^{a}	0.94±0.03^{bc}	10.43±0.29^{a}	172.00±25.87^{d}	160.20±2.30^{d}	2404.00±114.00^{a}
0.1%PE+LHMs	4.32±0.06^{a}	11.45±0.37^{b}	26.97±1.94^{abc}	0.14±0.00^{a}	0.81±0.17^{c}	10.27±0.25^{a}	166.00±14.00^{d}	166.65±16.05^{d}	1918.50±20.50^{d}
HHMs	4.43±0.16^{a}	11.45±0.93^{b}	26.30±1.05^{c}	0.15±0.01^{a}	1.17±0.20^{c}	9.83±0.30^{a}	189.00±22.00^{cd}	309.75±3.35^{e}	1945.33±90.43^{bc}
0.05%PE+HHMs	4.42±0.16^{a}	11.89±0.67^{ab}	29.60±2.34^{a}	0.15±0.01^{a}	0.79±0.07^{b}	10.27±0.44^{a}	218.00±15.00^{bc}	130.20±8.20^{b}	2021.00±140.76^{bc}
0.1%PE+HHMs	4.47±0.19a	12.85±0.22a	26.07±0.95c	0.15±0.01a	1.47±0.21a	10.03±0.62a	222.50±19.50bc	560.75±16.75a	1998.00±178.00^{bc}

Note: Data in the table are presented as mean ± standard deviation (Mean ± SD, n = 3). Different lowercase letters within the same column indicate significant differences among different treatments at the 0.05 level (Duncan's multiple range test).

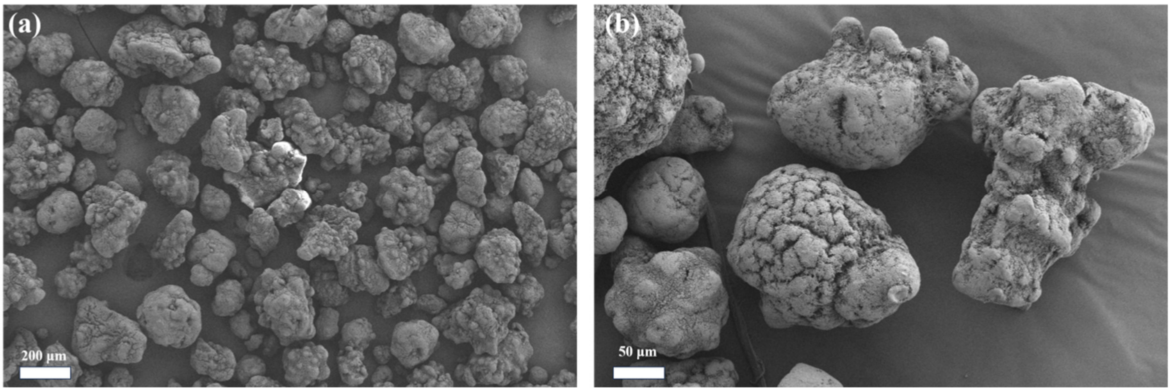

Figure S1. Scanning electron microscopy (SEM) micrographs of polyethylene (PE) samples at different magnifications. Images were acquired on a Zeiss SEM in secondary electron mode (SE2 detector) at an accelerating voltage (EHT) of 5.00 kV. Magnifications: (a) 50× with a scale bar of 200 µm, and (b) 200× with a scale bar of 50 µm.

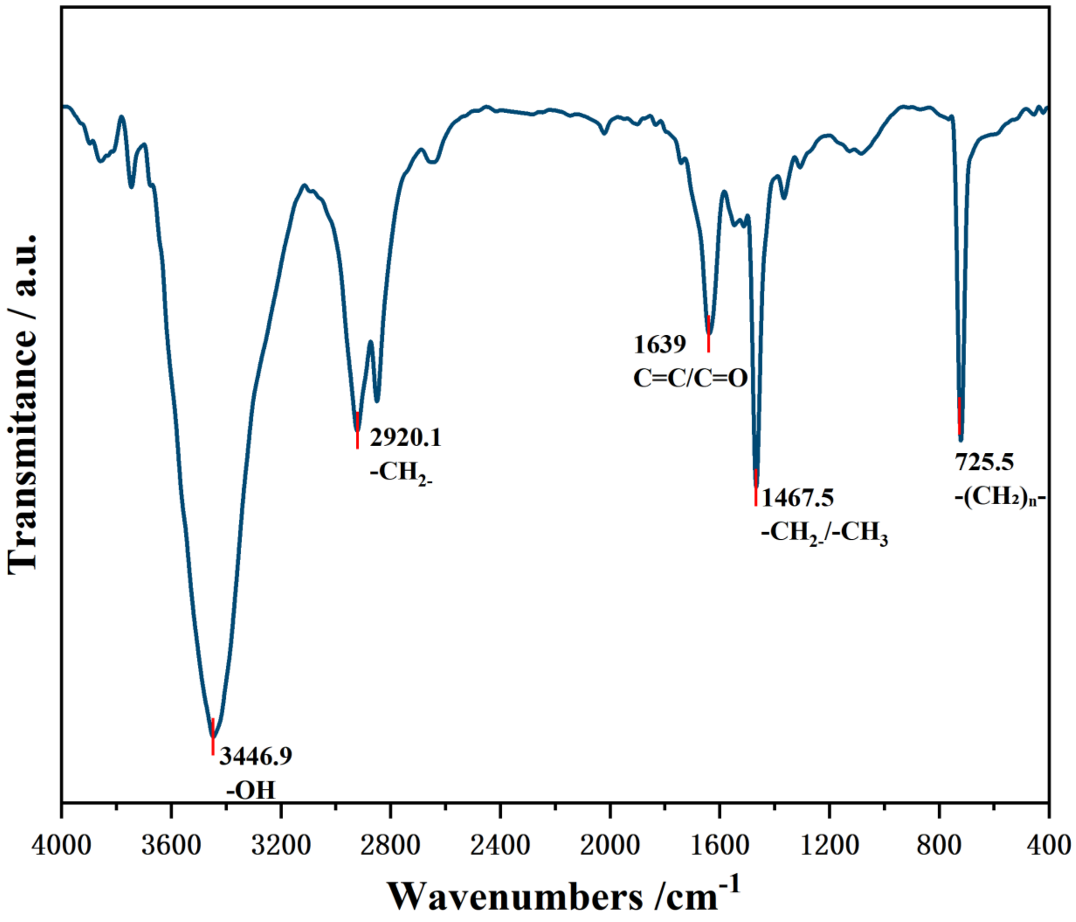

Figure S2. FTIR spectrum of the polyethylene (PE) microplastics used in the study.acquired in transmittance mode (4000–400 cm^{-1}, resolution = 4 cm^{-1}, 16 scans for both sample and background).The characteristic absorption bands at 2920.1 cm^{-1} (-CH_2-

asymmetric C-H stretching), 1467.5 cm^{-1} (-CH_2-/-CH_3 C-H bending), and 725.5 cm^{-1} (-$(CH_2)_n$-rocking vibration, $n \geq 4$) confirm the typical chemical structure of polyethylene. The weak band at 1639 cm^{-1} is attributed to C=C/C=O stretching, indicating minor surface oxidation, and the broad absorption at 3446.9 cm^{-1} corresponds to O-H stretching, likely from adsorbed moisture on the particle surface.
